# P278: Evaluating a new infection prevention and control programme (IPCP): tool development and use in the Republic of Kiribati

**DOI:** 10.1186/2047-2994-2-S1-P278

**Published:** 2013-06-20

**Authors:** P-A Zimmerman, H Yeatman, M Jones, H Murdoch

**Affiliations:** 1 School of Nursing and Midwifery, Griffith University, Southport, Australia; 2 Faculty of Health and Behavioural Sciences, Wollongong, Australia; 3 Faculty of Commerce, University of Wollongong, Wollongong, Australia; 4 Ministry of Health, Tarawa, Kiribati

## Introduction

IPCP audit or evaluation tools reported in the literature to be rigorous and validated have been found to focus on certain infection prevention and control activities such as hand hygiene and antibiotic stewardship. Less available are holistic tools for the evaluation of IPCPs.

## Objectives

This paper details the development and trialing of an infection prevention and control programme evaluation (IPCPE) tool for use in a low- and middle-income (LMI) healthcare setting, the Republic of Kiribati, during 2010. The IPCPE was developed to collect information on a number of aspects, identified by the World Health Organization (WHO), as necessary for inclusion as core components of a comprehensive IPCP.

## Methods

After reviewing the literature validated IPCP audit or evaluation instruments were considered adaptable to the LMI country setting: 1) the Infection Control Nurses Association’s (ICNA), and 2) *Audit tools for monitoring infection control standards* and the *Nosocomial Infection Program Rapid Evaluation Guide*, produced for use in LMI countries by the Pan American Health Organization/Regional Office of the WHO (PAHO). Each of these tools had specific but complementary deficits. They were combined to create a new evaluation document, which was suitable for assessment of comprehensive IPCP in LMI countries.

Experts in infection prevention and control with experience in LMI country healthcare settings, and a regional expert from Fiji (also an LMI country), pre-piloted and confirmed the validity of the IPCPE instrument. A pilot of the IPCPE was conducted in person by the authors in Kiribati.

## Results

A new IPCPE tool was created and tested in a LMI country setting. The Kiribati IPCP demonstrated a minimum level of compliance (75%) with the activity standards set out in the IPCPE.

## Conclusion

The development of the IPCPE and its application to evaluate the Kiribati IPCP, provide valuable insights into the status of a recently adopted programme in a LMI country setting.

## Competing interests

None declared

